# Treatment of chronic lateral ankle instability: a modified broström technique using three suture anchors

**DOI:** 10.1186/1749-799X-4-41

**Published:** 2009-12-02

**Authors:** Xinning Li, Timothy J Lin, Brian D Busconi

**Affiliations:** 1 University of Massachusetts Medical Center, Division of Sports Medicine, Dept of Orthopaedic Surgery, Room S4-827, 55 Lake Avenue North, Worcester, MA 01655, USA; 2 University of Massachusetts Medical School, Worcester, MA 01655, USA

## Abstract

Ankle sprains are very common injuries seen in the athletic and young population. Majority of patients will improve with a course of rest and physical therapy. However, with conservative management about twenty percent of all patients will go on to develop chronic lateral ankle instability. This manuscript describes our detailed surgical technique of a modification to the original Broström procedure using three suture anchors to anatomically reconstruct the lateral ankle ligaments to treat high demand patients who have developed chronic lateral ankle instability. The rationale for this modification along with patient selection and workup are discussed. Both the functional outcomes at the two year follow up along with the complications and the detailed postoperative rehabilitation protocol for the high demand athletes are also presented. This modified Broström procedure is shown in both illustrative format and intra-operative photos.

## Background

Ankle sprains are common injuries seen in the young and athletic population with majority of the cases involving the lateral ligamentous complex [1-3]. Much of the literature has been written about the operative and non-operative treatment of severe lateral ankle sprains and the possible sequelae of chronic instability of the ankle [4-14]. A majority of the patients will improve following a treatment protocol involving a period of rest and physical therapy. However, it has been noted in previous studies that as many as twenty percent of patients will have chronic symptomatic ankle instability [5,15,16]. The treatment of chronic ankle instability in patients who have failed a course of supervised and aggressive physical therapy, poses a challenge to the orthopedic surgeon. Many different techniques have been described in the operative treatment for chronic lateral ankle instability that involves either anatomic or non-anatomic repairs (tenodesis) [13,14,17-20]. Broström described an anatomic primary repair technique in 1966 [21] and Gould subsequently modified this technique by advancing the extensor retinaculum to reinforce the repair [22].

This manuscript describes another variant to the Gould modified Broström repair using three suture anchors. This technique involves anatomic reconstruction of both the Anterior Talo-fibular Ligament (ATFL) and Calcaneal Fibular Ligament (CFL). A third suture anchor is also used proximal to the ATFL insertion to reinforce the repair anatomically.

## Surgical Technique

**Figure 1:**
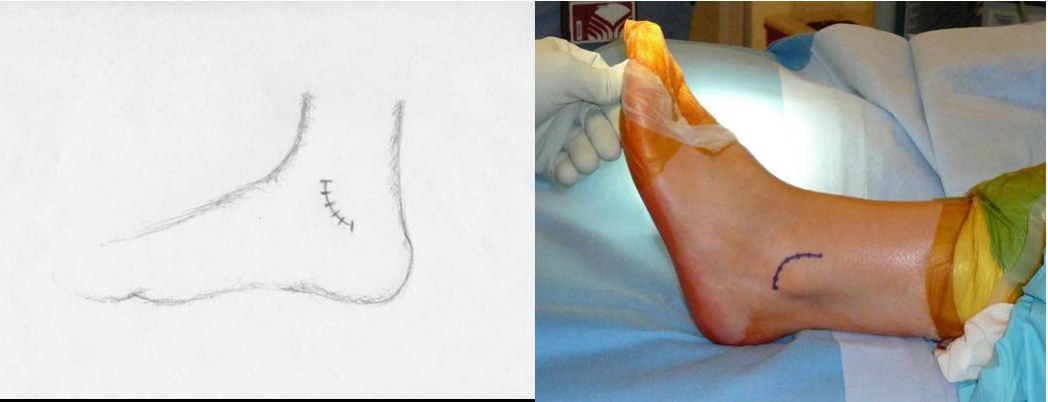
**A 2 inch curvilinear incision is made just anterior to the lateral malleoli with a #15 blade**. Care is taken to identify and avoid the sural nerve.

**Figure 2:**
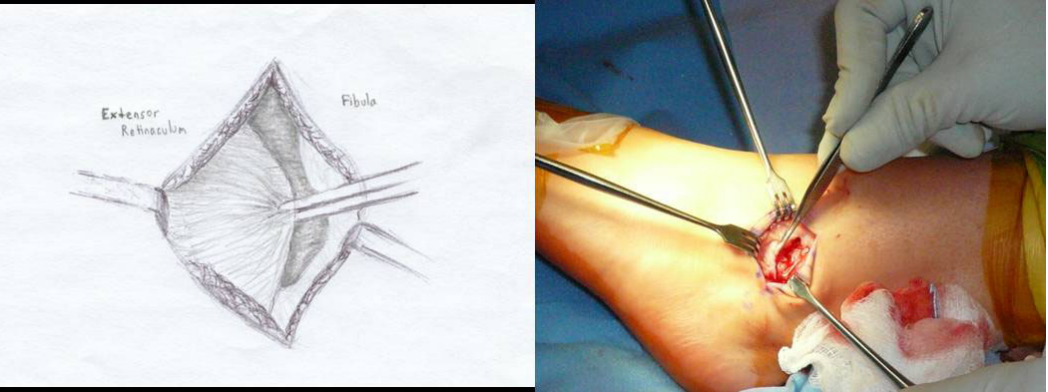
**The proximal edge of the inferior extensor retinaculum is identified and carefully dissected then mobilized for advancement later in the procedure**.

**Figure 3:**
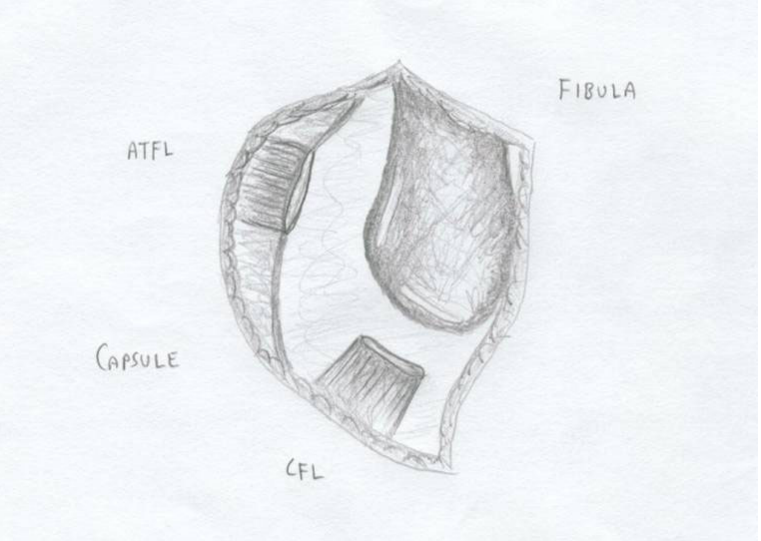
**The lateral ankle capsule was then identified along with the remnants of the anterior talofibular ligament**. The calcaneofibular ligament (CFL) can be identified at the tip of the distal fibula with inferior retraction of the peroneal tendons.

Each patient received general anesthesia with a peroneal nerve block. The patient was placed in a supine position with a towel bump under the ipsilateral buttock. A thigh tourniquet was utilized during the operation to control bleeding (250 mmHG). Once the patient was anesthetized, their ankle was evaluated for baseline range of motion and laxity. Next, a two inch curvilinear incision was made anteriorly over the lateral malleoli with a #15 blade (Figure 1). Care is taken to identify and avoid the sural nerve. The proximal edge of the inferior extensor retinaculum was then identified, carefully dissected and mobilized (Figure 2). The lateral ankle capsule was then identified along with the remnants of the anterior talofibular ligament. The calcaneofibular ligament (CFL) can be identified at the tip of the distal fibula with inferior retraction of the peroneal tendons (Figure 3). We also inspected the peroneal tendons for tears at this time. The capsule was then divided from the distal fibula and extended about one centimeter proximally via sub periosteal elevation with a #15 blade.

**Figure 4:**
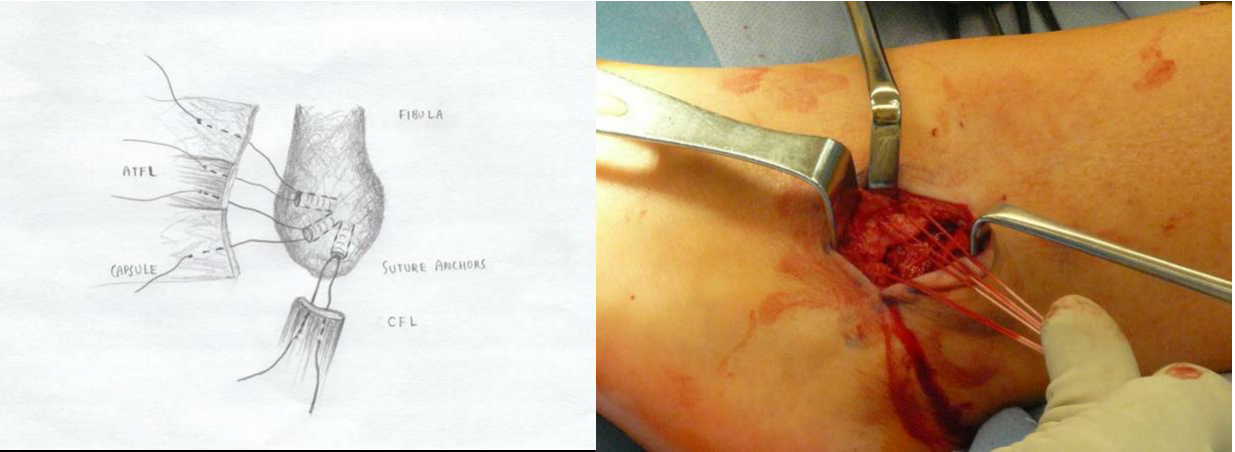
**Two suture anchors placed at the footprint of the ATFL and CFL insertion site**. A third suture anchor is placed approximately 1 cm above the ATFL insertion site to reinforce the repair. The orientation of the sutures from each anchor is also illustrated.

**Figure 5:**
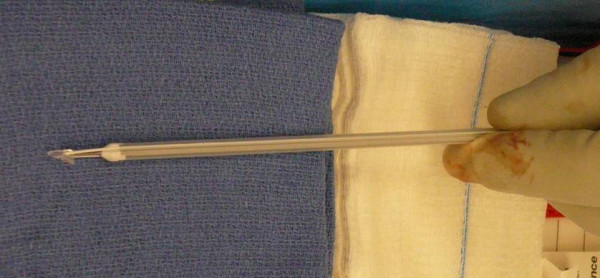
**Mitek - 4.1 × 6.0 mm with #2 Panacryl absorbable sutures used in our procedure**.

The lateral ankle gutter and the lateral talar dome were also inspected for loose bodies and osteochondral injuries. Next, the distal tip if the fibula at the ATFL and CFL footprint was debrided with a curette and surgical blade, then a burr was used to lightly decorticated the bone in order to provide a bleeding bony bed for healing and suture anchor placement. Drill holes were then made at the ATFL and CFL insertion site. A Panalok (Mitek) panacryl suture anchor was placed at the anatomic foot print of each, the ATFL and CFL, respectively in the distal fibula. A third Panalok (Mitek) suture anchor was inserted approximately 1 cm above the ATFL insertion with the same drill hole technique (Figure 4). Care was taken to make sure that the drill hole tunnels did not intersect with each other. Figure 5 shows the suture anchor (Mitek - 4.1 × 6.0 mm with #2 Panacryl absorbable suture) that we utilized for our procedure. The suture from anchor #1 was placed into the CFL in a horizontal mattress fashion. The inferior limb of anchor #2 was placed into the capsule just inferior to the ATFL, the superior limb (Anchor #2) and the inferior limb of anchor #3 was placed into the ATFL ligament using a horizontal mattress. Lastly, the superior limb of suture #3 was place above the ATFL into the capsule. Please see Figure 4 for the exact orientation of the sutures.

**Figure 6:**
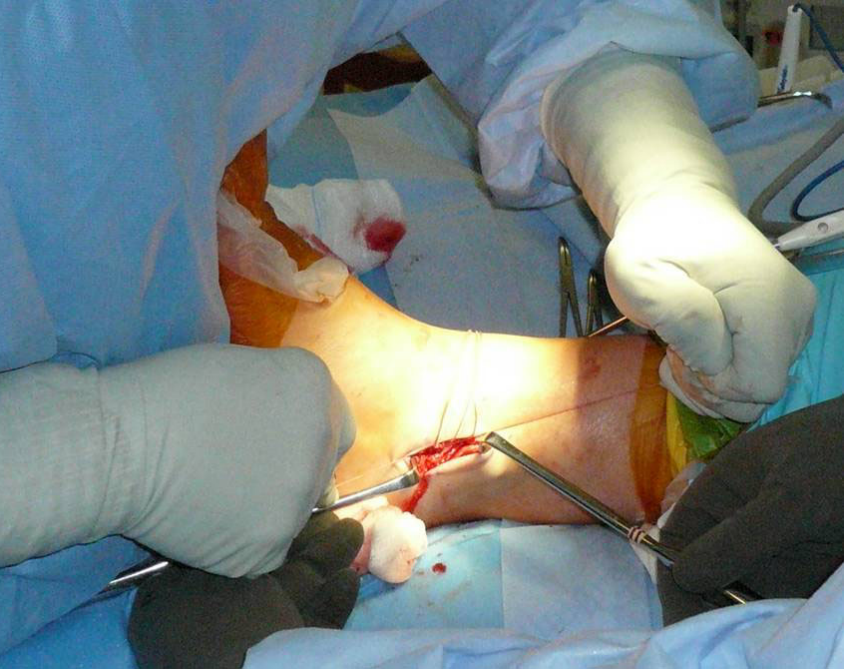
**The foot was held in neutral dorsiflexion and slight eversion when the sutures from the 3 anchors were tied down to further reinforce our repair**.

**Figure 7:**
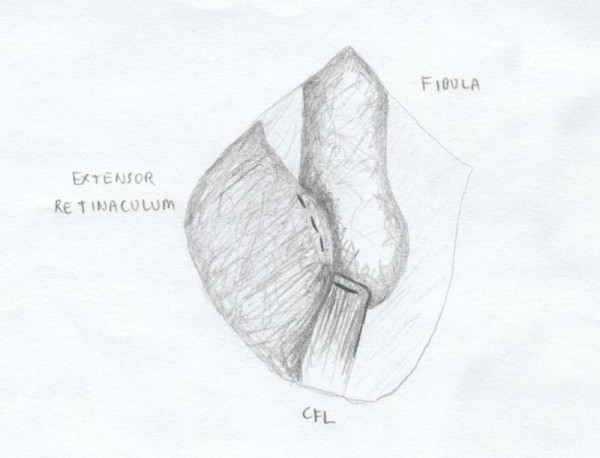
**The extensor retinaculum was then advanced and repaired to the periosteum of the distal fibula**.

**Figure 8:**
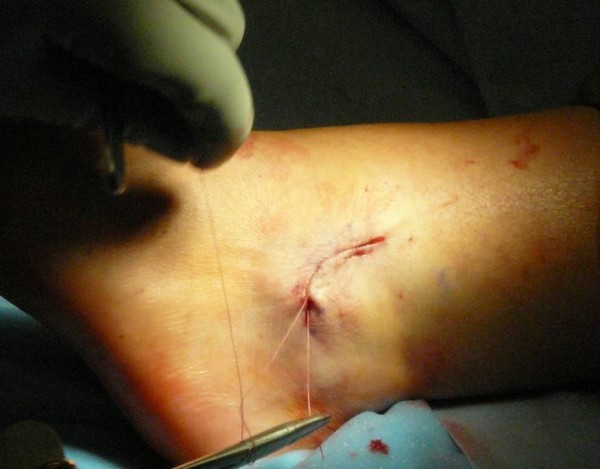
**The incision was closed subcutaneously using 2-0 vicryl sutures**.

Next, the remnants of the ATFL and CFL along with a capsular-periosteal flap were tied down to the three suture anchors with the foot at neutral dorsiflexion and slight eversion (Figure 6). The extensor retinaculum was then advanced and repaired to the periosteum of the distal fibula (Figure 7) to reinforce our repair with interrupted 0 vicryl sutures (this is also done with the foot in neutral dorsiflexion and slight eversion. Lastly, the skin was closed subcutaneously using 2-0 vicryl sutures (Figure 8), followed by 4-0 nylon interrupted stitches. Wound was dressed with xeroform and sterile 4 × 4 kurlex and webrile.

## Post Operative Protocol

Post-operatively, all patients were placed in a well-padded posterior and sugar-tong splint with the foot in neutral and slight eversion with non weight bearing instructions until their follow-up visit in 10 to 14 days. Toe range of motion was encouraged in order to diminish venous stasis. Aspirin of 325 mg PO daily for 14 days was also prescribed for each patient. At the first postoperative follow-up visit, ankle incision was inspected and the stitches were removed. The patients were then placed in a short-leg walking cast for the next 2 weeks. Protected and progressive weight bearing was allowed over the following 2 weeks. During weeks 4 to 6, patients were then placed in a protective ASO (ankle support orthosis) brace and started on gentle active assisted range of motion of the ankle. Proprioception and strength training were also started during the 6 to 8 week interval with plyometrics starting at 8 to 12 weeks. Patients were allowed to return to sports or normal activities without any limitations shortly thereafter week 16. If the patient is a highly competitive athlete, then the post operative protocol was modified to include straight running and functional activities at about the 12^th ^post operative week. Sports specific drills and cutting activities were allowed after week 16.

## Discussion

The majority of lateral ankle ligament injuries will resolve with non-operative care. Konradsen, et al. have showed that eighty percent of their patients with lateral ankle ligament injuries improved when treated with a course of supervised rehabilitation specifically aimed at proprioceptive and strength training with a seven year follow up [9]. However, there are still about twenty percent of patients that will fail conservative management and go onto develop chronic instability. Both anatomic and non-anatomic techniques have been utilized to treat chronic lateral ankle instability in the literature.

The rationale for this particular modification to the original Broström technique is the improved surgical versatility of utilizing suture anchors for the repair of the ATFL, CFL, and the anterior capsule. First, the placement of the suture anchor at the footprints of the ATFL and CFL allows the surgeon to address each of the ligaments individually and the ability to repair them anatomically. Secondly, using anchors with sutures that are tied to the distal aspects of the ligaments will allow the surgeon the ability to better tighten the lateral ankle structures in comparison to primary repair. This particular technique also adds a third suture anchor approximately 1 cm above the ATFL insertion site, which is used to tighten the confluence of the proximal aspect of the ATFL ligament with the lateral ankle capsule. This is an important step because in patients with lateral ankle instability and tear of the ATFL, sometimes it is very difficult to distinguish the margin of the ATFL with the lateral capsule. Thus the addition of the third suture anchor allows further anatomic reinforcement of the repair without compromising ankle motion. Furthermore, the suture and the anchor used are absorbed over time, thus leaving no hardware in the ankle joint.

Our patient selection for this procedure is chronic lateral ankle instability refractory to at least a 6 months course of formal physical therapy. Both the talar tilt and anterior drawer stress radiographs are done in clinic with a mini fluoroscopy to document lateral instability. It is extremely important to compare the stress views with the contra-lateral ankle as some patients may have congenital laxity. Magnetic resonance imaging is also ordered on every patient to evaluate for intra-articular pathology and condition of the ATFL/CFL ligaments. Anyone with fractures, significant varus mal-alignment, severe osteoarthritis of the ankle, osteochondral dissecans lesions of the talus, and previous failed lateral ankle ligamenteous repair or reconstruction are not candidates for this procedure. Important points to remember during surgery include 1) meticulous elevation of the extensor retinaculum to leave a cuff of tissue for advancement, 2) careful and accurate periosteal dissection of the capsule off the fibula in order to preserve adequate length for repair, 3) always evaluate the intra-articular aspect of the ankle joint looking for loose bodies or OCD lesions, 4) when repairing the CFL, pay attention not to incorporate the peroneal tendon into the repair, and 5) if the CFL or ATFL is significantly shortened or not repairable, our bailout procedure of choice is an allograft reconstruction of both the ATFL and CFL. Furthermore, the most essential components to the success of this procedure is to tighten the three suture anchors as well as the extensor retinaculum with the patient's foot in neutral and slight eversion (Figure 7) which will further tighten up the lateral instability.

This technique was utilized by the authors in a series of high demand athletes with chronic lateral ankle instability and was able to return 94% of the patients to their previous sports activity level as demonstrated by the Tegner score. Also the average Karlsson ankle functional score was 92 +/- 5.2 and 95 +/- 3.1 at the one and two year post operative time frame, respectively. Only 3 patients out of 52 (6%) had a decrease in range of motion of greater than 5 degrees at the two year post operative follow up and there was no loss in subtalar motion. The major complication rate included a 6% re-rupture rate (3/53 patients) past the 1 year post operative period due to traumatic injuries in competition with no neurological injuries. We had several superficial wound infections that were treated and resolved with a course of oral antibiotics. Also a few patients had persistent ankle swelling, however, all of them resolved at the 6 months follow-up visit [23].

## Conclusion

Operative management of chronic lateral ankle instability using anatomic technique with three suture anchors will provide a stable fixation for patients with chronic lateral ankle instability refractory to conservative management to return to their normal functional activity level. This modification also provides the surgeon with another alternative technique to the Broström repair.

## Competing interests

The authors declare that they have no competing interests.

## Authors' contributions

XL and BB have contributed to the conception/design, data collection/interpretation, and drafting/revising of the manuscript. XL and TL created the illustrations and intra-operative photos used in this manuscript. All authors approved the final manuscript.
